# New fluorescence spectroscopic method for the simultaneous determination of alkaloids in aqueous extract of green coffee beans

**DOI:** 10.1186/s13065-018-0431-4

**Published:** 2018-05-11

**Authors:** Hagos Yisak, Mesfin Redi-Abshiro, Bhagwan Singh Chandravanshi

**Affiliations:** Department of Chemistry, College of Natural Sciences, Addis Ababa University, P.O. Box 1176, Addis Ababa, Ethiopia

**Keywords:** Fluorescence spectroscopy, UV–VIS spectroscopy, Green coffee beans, Alkaloids, Caffeine, Theobromine, Trigonelline, Water extract, Ethiopia

## Abstract

**Background:**

There is no fluorescence spectroscopic method for the determination of trigonelline and theobromine in green coffee beans. Therefore, the objective of this study was to develop a new fluorescence spectroscopic method to determine the alkaloids simultaneously in the aqueous extract of green coffee beans.

**Results:**

The calibration curves were linear in the range 2–6, 1–6, 1–5 mg/L for caffeine, theobromine and trigonelline, respectively, with R^2^ ≥ 0.9987. The limit of detection and limit of quantification were 2, 6 and 7 µg/L and 40, 20 and 20 µg/L for caffeine, theobromine and trigonelline, respectively. Caffeine and trigonelline exhibited well separated fluorescence excitation spectra and therefore the two alkaloids were selectively quantified in the aqueous extract of green coffee. While theobromine showed overlapping fluorescence excitation spectra with caffeine and hence theobromine could not be determined in the aqueous extract of green coffee beans. The amount of caffeine and trigonelline in the three samples of green coffee beans were found to be 0.95–1.10 and 1.00–1.10% (w/w), respectively. The relative standard deviations (RSD ≤ 4%) of the method for the three compounds of interest were of very good. The accuracy of the developed analytical method was evaluated by spiking standard caffeine and trigonelline to green coffee beans and the average recoveries were 99 ± 2% for both the alkaloids.

**Conclusions:**

A fast, sensitive and reliable fluorescence method for the simultaneous determination of caffeine and trigonelline in the aqueous extract of green coffee beans was developed and validated. The developed method reflected an effective performance to the direct determination of the two alkaloids in the aqueous extract of green coffee beans.

## Background

**Fig. 1:**
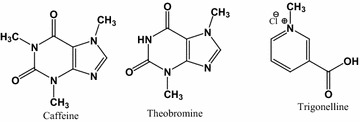
Structures of the three coffee alkaloids studied

Coffee is one of the most widely consumable beverages around the globe nowadays [1–3]. The most commercialized coffee species universally are *Coffea arabica* and *Coffea canephora* commonly known as *arabica* and *robusta* varieties [4, 5]. The *arabica* variety which is higher in cost than *robusta* variety due to its lower bitterness, better aroma and flavor is more prized by consumers [6]. Coffee is the principal source of bioactive compounds that mainly comprises alkaloids classified as methylxanthines (caffeine, theobromine and theophylline) and trigonelline [4] and the structures of these coffee alkaloids are shown in Fig. 1. The two types of alkaloids are derived from nucleotides. These are purine alkaloids like caffeine (1,3,7-trimethylxanthine) and theobromine (3,7-dimethylxanthine) as well as pyridine alkaloid, trigonelline (1-methylnicotinic acid) [7].

Caffeine and theobromine are essentially found in coffee beans, tea leaves, cacao beans, cola nuts and mate leaves [8, 9]. But caffeine is the predominant alkaloid in coffee. Even though trigonelline which is the second class of alkaloid occurs in coffee, barley, corn, onion, pea, soybean and tomato, it is the second most abundant alkaloid in coffee [9]. To determine the amount of coffee constituents like these of methylxanthines (caffeine and theobromine) as well as trigonelline in particular and to examine the quality, aroma and properties of coffee in general, developing sensitive, precise and accurate analytical method is worthwhile [6, 10]. This is due to the fact that methylxanthines and trigonelline do have desirable contribution to the typical flavor and aroma of coffee beverage [9] besides to their merit to human and animal health. For instance, methylxanthines (caffeine and theobromine) were reported to inhibit the elevation of body fat percentage in the developmental stage of rats, improve blood microcirculation and cardiovascular activities, use in the treatment of congestive heart failure and anginal syndrome, reduce the risk of coronary heart disease and stroke, decrease type 2 diabetes mellitus incidence and attribute relevant anti-cancer actions and potential [11]. In addition, caffeine is recognized as a stimulant to the central nervous system and is generally related with enhancement of alertness, learning capacity, relaxation, recreation, providing energy, decrease fatigue, performance enhancement, muscle relaxant when reasonably consumed [2, 12]. Theobromine also stimulates the central nervous system to a lower degree than caffeine [1], usually used as smooth muscle relaxant and also causes dieresis [13]. Trigonelline which is pyridine alkaloid derived from the methylation of the nitrogen atom of nicotinic acid does have hypoglycemic, hypolipidemic, sedative, anti-migraine, anti-bacterial, anti-viral, anti-tumor activities and is able to improve memory, hinder platelet aggregation [6] and anti-invasive activity against cancer cells [12].

The amount of alkaloids (caffeine, theobromine and trigonelline) in green coffee beans is influenced by numerous factors such as coffee variety, genetic properties of the cultivars, environmental factors (soil, altitude, sun exposure), climatic parameters (rainfall, temperature), maturity of the beans at harvest, harvesting method and agricultural practices (shade, pruning, fertilization) [14]. For instance, the amount of alkaloids found in green *arabica* coffee beans were reported in the range 0.87–1.38% (w/w), 0.0048–0.0094% (w/w) and 0.98–1.32% (w/w) for caffeine, theobromine and trigonelline, respectively [9] and another report revealed, 0.8–1.4% (w/w), 0.6–1.2% (w/w) for caffeine and trigonelline, respectively, while on the other hand, 1.7–4.0% (w/w), 0.3–0.9% (w/w) for the respective alkaloids in green *robusta* coffee beans [15]. However, the content of theobromine in coffee is considerably lower than caffeine and is hardly ever investigated [4].

Many analytical methods were reported for the determination of alkaloids in coffee such as ultraviolet visible spectroscopy [16, 17], Fourier transform near infrared spectroscopy [17, 18], high performance liquid chromatography [6, 9], electro analytical methods like voltammetry [19]. But the spectroscopic methods are getting more attention in the mean time due to their rapidity, cost effectiveness, simplicity, reliability and ability to measure multiple components without tedious sample preparation. In addition, these methods are vastly applicable to determine food composition because of their suitability on regular activities [20, 21]. Among the spectroscopic methods, UV–VIS spectroscopy is the most widely used for the determination of caffeine in different types of coffee samples. However, UV–VIS spectroscopy is not applicable for the direct determination of caffeine in aqueous extract of coffee beans and hence requires selective extraction of caffeine into organic solvents like dichloromethane [16, 17]. Furthermore, there is no any report for the determination of trigonelline and theobromine in green coffee beans by UV–VIS spectroscopy. Although the FT-IR spectroscopy can be used for the direct determination of caffeine in aqueous extract of coffee beans, however, it is less sensitive than UV–VIS spectroscopy. Besides, there is no any report for the determination of trigonelline and theobromine in green coffee beans by FT-IR spectroscopy. The literature survey also revealed that there is no fluorescence spectroscopic method for the simultaneous determination of alkaloids in green coffee beans. Therefore, the objective of this study was to develop a new fluorescence spectroscopic method to determine alkaloids simultaneously in the aqueous extract of green coffee beans.

## Experimental

### Chemicals and samples

The chemicals and reagents used were of analytical grade. Caffeine (J.T. Baker Chemical Company (Phillipsburg, USA), theobromine (Sigma-Aldrich, Italy) and trigonelline hydrochloride (Sigma-Aldrich, Switzerland) were used as received. Three *arabica* green coffee bean samples were collected from the Southern Nations, Nationalities and Peoples Region (SNNPR), Ethiopia, specifically from Abosto (Sidama), Gedeo zone and Wendogenet (Sidama). Distilled water was used as a solvent throughout the study.

### Instruments and apparatus

The fluorescence emission and excitation spectra of the standards and samples of the compounds of interest (caffeine, theobromine and trigonelline) were obtained using 1 cm path length with four side transparent quartz cuvette and recorded on Perkin Elmer Hitachi Spectrofluorimeter (Flouromax-4, Spectrofluorimeter, USA) with a xenon lamp source interfaced to a computer supplied to origin data manager software. The excitation response of the standard solutions was also scanned by Perkin Elmer UV–VIS–NIR Spectrophotometer. The light source for the Perkin Elmer UV–VIS–NIR Spectrophotometer was a deuterium discharging lamp for the UV range and a tungsten-halogen lamp for visible range. Hence, a double beam UV–VIS–NIR Spectrometer, Perkin Elmer Lambda 950 (Perkin Elmer, Llantrisant, CF728YW, and UK) which was operated by Perkin Elmer, UV win Lab software was used. All the experimental data were analyzed by using origin software (version 6).

### Preparation of standard alkaloid solutions

Standard solutions of caffeine, theobromine and trigonelline were prepared by weighing 0.100 g of the standards separately on an electronic balance and dissolved in 400 mL distilled water in separate 500 mL beakers. Solubility was facilitated by magnetic stirrer with hot plate (~ 40 °C) for caffeine and theobromine since they are slightly soluble in water but trigonelline is completely soluble in water without applying stirrer. The solution was allowed to cool down to the room temperature (22 °C) for the solutions of the two alkaloid (caffeine and theobromine) standards. The solution was transferred to 1000 mL separate volumetric flasks and diluted with distilled water up to the mark. The intermediate solutions for the three alkaloid standards were prepared by diluting 25 mL of the stock solution to 100 mL in separate volumetric flasks to produce 25 mg/L concentration of the respective alkaloids.

The working standard solutions for calibration were prepared by diluting 2000, 3000, 4000, 5000 and 6000 µL, 1000, 2000, 3000, 4000 and 6000 µL, 1000, 2000, 3000, 4000 and 5000 µL of caffeine, theobromine and trigonelline intermediate solution, respectively to 25 mL with distilled water to get concentrations of 2, 3, 4, 5 and 6 mg/L, 1, 2, 3, 4, and 6 mg/L, 1, 2, 3, 4, and 5 mg/L of caffeine, theobromine and trigonelline, respectively. The working standard solutions were run in triplicates in the suitable spectral ranges selected for this study to collect the desired data.

### Preparation of green coffee beans samples

The three green coffee bean samples were ground using mortar and pestle and screened via 300 μm sieve in order to have uniform texture. A 0.2 g of the ground green coffee powder was dissolved in 40 mL of distilled water. The solution was stirred for one and half hour using magnetic stirrer over hot plate (~ 40 °C) to dissolve the alkaloids of green coffee powder. The solution was filtered through Whatman filter paper to separate the insoluble particles from the solution. The filtrate (clear solution) was taken for quantitative determination of coffee alkaloids by the developed method (fluorescence).

### Determination of limit of detection (LOD) and limit of quantification (LOQ)

The limit of detection (LOD) and limit of quantification (LOQ) of the developed method (fluorescence) were determined with respect to the alkaloids by preparing 1 mg/L of standard solutions and filled in the quartz cuvette followed by rinsing ten times using the solvent (distilled water) and then finally the quartz cuvette was filled by distilled water (solvent) and scanned ten times in the selected range by adjusting the accumulation scan at 20, slit width 15 nm to collect data. The LOD and LOQ were computed three times and ten times, respectively, of the standard deviation of the background signal from ten measurements divided by the slope of the calibration equation.

### Determination of caffeine, theobromine and trigonelline in aqueous extract of green coffee beans

To determine the amount of alkaloids in the aqueous extract of green coffee beans, calibration curves were established for each compounds of interest from the series of concentrations (2–6, 1–6 and 1–5 mg/L) of caffeine, theobromine and trigonelline standards, respectively. The fluorescence excitation spectra were recorded at the typical absorption band obtained around 275, 276 and 267 nm for caffeine, theobromine and trigonelline, respectively. The concentration of caffeine, theobromine and trigonelline in the aqueous extract of green coffee beans were determined from the respective calibration curves.

## Results and discussion

### Spectral characteristics of coffee alkaloid standards

**Fig. 2:**
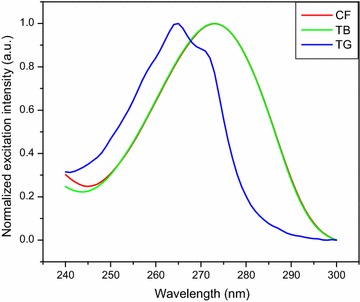
UV–VIS excitation spectra of coffee alkaloid standards dissolved in water. *CF* caffeine, *TB* theobromine, *TG* trigonelline

**Fig. 3:**
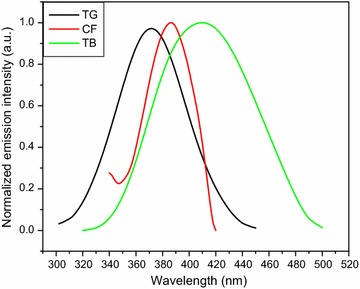
Fluorescence emission spectra of coffee alkaloid standards dissolved in water. *TG* trigonelline, *CF* caffeine, *TB* theobromine

Caffeine, theobromine and trigonelline standard solutions were scanned by UV–VIS and fluorescence spectroscopic methods to determine their maximum excitation and emission wavelength. The alkaloid standards were scanned in the UV–VIS method over the free spectral range (200–400 nm) by fixing the lamp change at 319.20 nm, scan speed at 266.75 nm/min and slit width at 2 nm. The UV–VIS excitation and fluorescence emission spectra of the three alkaloids are shown in Figs. 2 and 3, respectively. The maximum UV–VIS excitation bands were obtained at 272.89 nm (caffeine), 272.73 nm (theobromine) and 264.59 nm (trigonelline). The spectral response of UV–VIS method indicated that the two alkaloids (caffeine and theobromine) were overlapped which cannot be differentiated by using this method as shown in Fig. 2. Besides, the standard solutions were also run by fluorescence method both in the excitation and emission spectral acquisition modes. Once, the maximum excitation spectral response of the alkaloids were collected from the UV–VIS method, the maximum emission spectral results were also collected from fluorescence method by running the standard solutions of the individual alkaloids over the range 340–420, 320–500 and 300–450 nm for caffeine, theobromine and trigonelline, respectively. The fluorescence emission spectral responses were found at 386, 410 and 370 nm for the respective alkaloids. Therefore, fluorescence method which is very sensitive and fast one was able to differentiate the three coffee alkaloids in the emission spectral acquisition mode by using water as a solvent. Hence, fluorescence method was selected and developed to determine the three coffee alkaloids in this study.

### Selection of working spectral acquisition mode and ranges for the three coffee alkaloids

To identify the maximum emission wavelength (λ_emi_) of the three alkaloids by the developed method (fluorescence) using distilled water as a solvent, systematic study was made by scanning the standard and sample solutions in the spectral acquisition emission mode of the method. The maximum emission wavelength was obtained at 386, 410 and 370 nm for caffeine, theobromine and trigonelline, respectively, which was far from Rayleigh and Raman scattering. To conduct quantitative determination of the alkaloids in the aqueous extract of green coffee beans using the newly developed method, the fluorescence excitation spectral acquisition mode was used. This was due to the reason that the emission spectral acquisition mode was highly exposed to fluctuation (correlation coefficients of calibration curves, R^2^ < 0.67 and the RSD > 15%) than the excitation spectral acquisition mode (correlation coefficients of calibration curves, R^2^ > 0.999 and the RSD ≤ 4%). Hence, all the standard and sample solutions were scanned in the fluorescence excitation spectral acquisition mode in the range 255–295, 260–290 and 245–286 nm for caffeine, theobromine and trigonelline, respectively, to construct calibration curves for the determination of the amount of alkaloids in the aqueous extract of green coffee beans using the calibration equation.

### Analytical characteristics

In fluorescence spectroscopic measurement, highly concentrated solutions are not suitable, because fluorescence is very sensitive method and needs low concentration as far as inner filter effect is occurred in which molecules of the solution as a whole is not uniformly exciting and then emitting. The standard and sample solutions were scanned in triplicate in the fluorescence excitation spectral acquisition mode within the required ranges by fixing the maximum emission wavelength at 386, 410, 370 nm, slit width at 15 nm and accumulation scan 20 for caffeine, theobromine and trigonelline, respectively, to collect the desired data throughout the entire study.

**Fig. 4:**
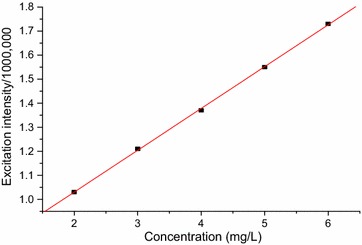
Graph of concentration vs maximum fluorescence excitation intensity of standard caffeine dissolved in water

**Fig. 5:**
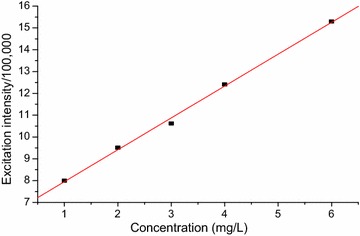
Graph of concentration vs maximum fluorescence excitation intensity for theobromine standard dissolved in water

**Fig. 6:**
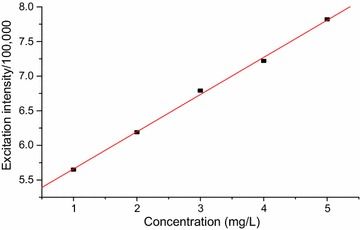
Graph of concentration vs maximum fluorescence excitation intensity for trigonelline standard dissolved in water

The calibration curves for the developed method (Figs. 4, 5 and 6) were linear with calibration equations of y = 0.174x + 0.682, y = 1.460x + 6.492 and y = 0.537x + 5.123, where, y designates fluorescence excitation intensity and x indicates concentration in mg/L. The linearity of the calibration curves were evaluated based on the magnitude of coefficient of determination (R^2^ = 0.9998, 0.9987 and 0.9990 for caffeine, theobromine and trigonelline, respectively).

**Fig. 7:**
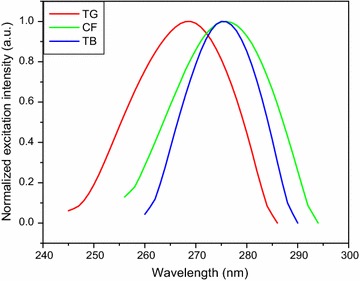
Fluorescence excitation spectra of coffee alkaloids in the aqueous extract of green coffee beans. *TG* trigonelline, *CF* caffeine, *TB* theobromine

The amount of caffeine and trigonelline in aqueous extract of green coffee beans were determined by diluting the sample solution [0.2 g in 40 mL solvent (distilled water)] 15 and 50 times and scanned the diluted solution by the fluorescence method to collect the required data and the concentration of the alkaloids were determined using the calibration equations. The percentage caffeine and trigonelline in the green coffee beans were calculated from the concentration of the alkaloids in the aqueous extract of green coffee beans. The results obtained from the triplicate measurements were 0.95–1.01% (w/w) for caffeine and 1.00–1.10% (w/w) for trigonelline which are comparable with the literature results reported in the ranges 0.87–1.38% (w/w) for caffeine and 0.98–1.32% (w/w) for trigonelline [9], 0.8–1.4% (w/w) for caffeine and 0.6–1.2% (w/w) for trigonelline [15], 0.90–1.10% (w/w) for caffeine [22], 0.80–1.40% (w/w) for caffeine and 0.60–1.20% (w/w) for trigonelline [23]. The results of the present study are given in Table 1. However, theobromine was not quantified in the green coffee beans because of its overlapping fluorescence excitation wavelength with that of caffeine as shown in Fig. 7. But it is possible to quantify theobromine in any real sample with lower amount (does not contain) of caffeine.

**Table 1:** Amount of alkaloids in green coffee beans obtained by fluorescence method

Origin of green coffee beans sample	Alkaloid in green coffee beans sample	Amount of alkaloid in green coffee beans sample (mean ± SD) %w/w
Abosto (Sidama)	Caffeine	0.95 ± 0.004
	Trigonelline	1.01 ± 0.01
Gedeo zone	Caffeine	1.01 ± 0.004
	Trigonelline	1.10 ± 0.01
Wendogenet (Sidama)	Caffeine	0.98 ± 0.02
	Trigonelline	1.03 ± 0.005

If green coffee beans contain smallest amount of caffeine (0.5% w/w as reported by Demissie et al. [16] and highest amount of theobromine (0.01% w/w as reported by Mehari et al. [9], the error involved in the measurement of caffeine in the presence of theobromine will be approximately 2%. Therefore, the maximum error that might occur in the determination of lowest amount of caffeine (0.5% w/w) in green coffee beans containing maximum amount of theobromine (0.01% w/w) will be 2% which is within the acceptable range. Hence, the interference of theobromine in quantification of caffeine can easily be ignored.

### Method validation

The validity of the developed fluorescence method for determining the alkaloids was evaluated in terms of the basic parameters; linearity, limit of detection (LOD), limit of quantification (LOQ), precision (% RSD) and accuracy (% recovery) done in triplicates. The calibration curves were linear over the range 2–6, 1–6 and 1–5 mg/L for caffeine, theobromine and trigonelline, respectively. The correlation coefficient (R^2^) was 0.9998, 0.9987 and 0.9990, respectively, for the alkaloids and revealed strong relationship among the concentration ranges.

The LOD and LOQ were 2, 6 and 7 µg/L and 40, 20 and 20 µg/L for caffeine, theobromine and trigonelline, respectively. The reproducibility of the method was evaluated by scanning the lower end of calibration curve’s concentration ten times and calculating the coefficient of variation or relative standard deviation (RSD) and the results found were 3, 3 and 4% for caffeine, theobromine and trigonelline, respectively. The accuracy of the developed analytical method was evaluated by spiking 0.2 mL of 2 mg/L caffeine and trigonelline standard solutions to 1 mL of the aqueous extract of green coffee beans and diluted to 15 and 50 mL, respectively. The results of recovery are given in Table 2. The basic parameters of the developed method are compared with the reported methods (Table 3). As can be seen from Table 3, the analytical parameters: LOD, LOQ, RSD, and percentage recovery of caffeine and trigonelline of the present method are better than most of the reported methods.

**Table 2:** Recovery results of caffeine and trigonelline by the developed fluorescence method

Origin of green coffee beans sample	Type of alkaloid in the sample	Amount of alkaloid in the sample before spiking (mg/L)	Amount of alkaloid added (mg/L)	Amount of alkaloid found after spiking (mg/L)	Recovery (%) (n = 3)
Abosto (Sidama)	Caffeine	67.1	2	69.1	102 ± 3
	Trigonelline	73.3	2	75.2	96.0 ± 3
Gedeo zone	Caffeine	59.3	2	61.2	94.0 ± 2
	Trigonelline	124	2	126	99.0 ± 2
Wendogenet (Sidama)	Caffiene	73.2	2	75.2	101 ± 2
	Trigonelline	93.2	2	95.2	102 ± 2

**Table 3:** Comparison of LOD, LOQ, RSD and percentage recovery of the developed method with the literature methods

Compound	LOD (µg/L)	LOQ (µg/L)	RSD (%)	Recovery (%)	Method	References
Caffeine	3610	10,940	1.7	95 ± 7	HPLC–DAD–MS	[4]
Theobromine	30	100	6.5	97 ± 3		
Trigonelline	3770	11,420	1.0	102 ± 2		
Caffeine	80	–	0.20–6.2	99 ± 3	HPLC–UV	[9]
Theobromine	140		2.8–9.1	103 ± 6		
Trigonelline	260		1.1–5.0	99 ± 5		
Caffeine	11.9	39.6	2.1–4.7	97 ± 4	LC–MS	[12]
Theobromine	–	–	–	–		
Trigonelline	36.4	121.5	3.2–3.5	140 ± 16		
Caffeine	2	40	3	99 ± 2	Fluorescence	This study
Theobromine	6	20	3	–		
Trigonelline	7	20	4	99 ± 2		

## Conclusions

A sensitive, rapid and cost effective fluorescence method was developed for the simultaneous determination of alkaloids in the aqueous extract of green coffee beans. Caffeine and trigonelline were determined but not theobromine due to its overlapping fluorescence excitation response with that of caffeine though its interference is negligible. The developed method revealed comparable recoveries and reproducibility with the reported results of chromatographic methods (LC–MS, HPLC–UV and HPLC–DAD–MS). The detection and quantification limits of the developed method with respect to the analytes were lower compared to the reported methods. This confirms the better sensitivity of the developed method which can make it to be applicable for the routine analysis of food containing coffee alkaloids.
